# Impairment of arbitration between model-based and model-free reinforcement learning in obsessive–compulsive disorder

**DOI:** 10.3389/fpsyt.2023.1162800

**Published:** 2023-05-26

**Authors:** Zhongqiang Ruan, Carol A. Seger, Qiong Yang, Dongjae Kim, Sang Wan Lee, Qi Chen, Ziwen Peng

**Affiliations:** ^1^Guangdong Key Laboratory of Mental Health and Cognitive Science, School of Psychology, Center for Studies of Psychological Application, South China Normal University, Guangzhou, China; ^2^Department of Psychology, Colorado State University, Fort Collins, CO, United States; ^3^Affective Disorder Center, Affiliated Brain Hospital of Guangzhou Medical University (Guangzhou Huiai Hospital), Guangzhou, China; ^4^Department of AI-based Convergence, College of Engineering, Dankook University, Yongin, Republic of Korea; ^5^Department of Bio and Brain Engineering, Program of Brain and Cognitive Engineering, Korea Advanced Institute of Science and Technology (KAIST), Daejeon, Republic of Korea; ^6^School of Psychology, Shenzhen University, Shenzhen, China; ^7^Key Laboratory of Brain, Cognition and Education Sciences, Ministry of Education, Guangzhou, China; ^8^Department of Child Psychiatry, Shenzhen Kangning Hospital, Shenzhen University School of Medicine, Shenzhen, China

**Keywords:** obsessive-compulsive disorder, goal-directed system, habitual system, model-based reinforcement learning, model-free reinforcement learning, arbitration system

## Abstract

**Introduction:**

Obsessive–compulsive disorder (OCD) is characterized by an imbalance between goal-directed and habitual learning systems in behavioral control, but it is unclear whether these impairments are due to a single system abnormality of the goal-directed system or due to an impairment in a separate arbitration mechanism that selects which system controls behavior at each point in time.

**Methods:**

A total of 30 OCD patients and 120 healthy controls performed a 2-choice, 3-stage Markov decision-making paradigm. Reinforcement learning models were used to estimate goal-directed learning (as model-based reinforcement learning) and habitual learning (as model-free reinforcement learning). In general, 29 high Obsessive–Compulsive Inventory-Revised (OCI-R) score controls, 31 low OCI-R score controls, and all 30 OCD patients were selected for the analysis.

**Results:**

Obsessive–compulsive disorder (OCD) patients showed less appropriate strategy choices than controls regardless of whether the OCI-R scores in the control subjects were high (*p* = 0.012) or low (*p* < 0.001), specifically showing a greater model-free strategy use in task conditions where the model-based strategy was optimal. Furthermore, OCD patients (*p* = 0.001) and control subjects with high OCI-R scores (H-OCI-R; *p* = 0.009) both showed greater system switching rather than consistent strategy use in task conditions where model-free use was optimal.

**Conclusion:**

These findings indicated an impaired arbitration mechanism for flexible adaptation to environmental demands in both OCD patients and healthy individuals reporting high OCI-R scores.

## 1. Introduction

Obsessive–compulsive disorder (OCD) is a chronic psychiatric disorder characterized by persistent, intrusive thoughts (obsessions) and repetitive, stereotyped behaviors (compulsions). Compulsive behaviors in OCD have been postulated as resulting from alterations in the instrumental behavioral control system, which includes two distinct, parallel systems of behavioral control: goal-directed and habitual (1, 2). Goal-directed control selects behavior based on a mental model of the world, which it uses to predict outcomes and adjust behaviors according to changes in the environment. Goal-directed control is highly flexible and forward-looking but is computationally expensive. In contrast, habitual control is based on previously learned stimulus–response relationships without consideration of the current environment. Habitual control is inflexible and retrospective but computationally highly efficient. In healthy individuals, the two systems work together to maximize beneficial choices while minimizing computational expense. Typically, in the early phases of learning in a novel environment, goal-directed control is used to develop a mental model of the environment and learn what behavioral choices are effective. As the environment stabilizes and the task becomes familiar, behavior gradually shifts to habitual control. If the environment changes, goal-directed control re-engages to adjust behavior. Researchers have argued that OCD is characterized by excessive reliance on habitual control and that obsessions and compulsions can be considered to be maladaptive habits (3). The shift to excessive use of habits in OCD could be due to one or a combination of several mechanisms: an increased strength of habit, decreased strength of goal-directed behaviors, or an impairment in a separate arbitration mechanism that selects which system controls behavior at each point in time (3, 4). We used a three-stage task with computational modeling to test the hypothesis that OCD is characterized by impairments in the third possible mechanism: arbitration between habitual and goal-directed control.

Computational neuroscientists have proposed that goal-directed and habitual behavioral control processes can be characterized by two reinforcement learning algorithms, namely model-based and model-free reinforcement learning, respectively (RL) (5–7). The model-based (MB) system builds an intrinsic model about state transitions in the decision-making process, taking into consideration state transition relationships (state prediction error, SPE) to make behavioral choices. In contrast, the model-free (MF) system learns the value of different behaviors *via* reward prediction error (RPE), based solely on prior experience with specific stimulus–response associations. More recently, researchers proposed that independent model-based and model-free reinforcement learning systems alone cannot fully account for human behavior (1). They have proposed that the two systems are subject to an arbitration system that weights the outputs of the two systems and controls when each is in charge of selecting behavior (8–11).

Previous research has consistently found increased use of MF and lower use of MB strategies in OCD (12–15), consistent with other findings of goal-directed and habitual system imbalances in patients with OCD (3, 16–20). When goal-directed and habitual learning systems were studied independently, studies typically found impairments in the goal-directed system in isolation or simultaneous with changes in the habitual system but typically not impairments in the habitual system in isolation. One functional magnetic resonance imaging (fMRI) study reported that the caudate nucleus and medial orbitofrontal cortex of OCD patients were more active during behavior acquisition, which may indicate that the bias toward habits in OCD is caused by changes in the goal-directed system (16). Another fMRI study found that during symptom provocation, activity in the goal-directed system (caudate nucleus, dorsolateral prefrontal cortex, and ventromedial prefrontal cortex) in OCD patients was weakened, while activity in the habitual system (putamen and auxiliary motor areas) was enhanced (18). Studies of intrinsic functional connectivity and anatomical connectivity within the neural systems known to underlie goal-directed and habitual behavior have shown decreased connectivity within the goal-directed system, and between the goal-directed and habitual systems, but not within the habitual system itself (21). In summary, previous research has found clear evidence for impairment in goal-directed behavior in OCD and activation changes in neural systems associated with goal-directed behavior, but it is unclear whether these impairments are due to a single system abnormality of the goal-directed system or due to an impairment in arbitration.

Research in this area has been limited due to the use of experimental paradigms that are incapable of isolating the goal-directed, habitual, and arbitration processes. Using traditional paradigms, habitual behavior is mainly inferred from the observed impairment of goal-directed behavior and lacks a clear independent operational definition. Therefore, using these tasks, it is difficult to precisely assess the independent contributions of each of the two systems (17, 22). We used a three-stage reinforcement learning task that was developed to study the behavioral and neural processes of arbitration (9). Computational modeling of trial-by-trial behavioral data gives estimates of each individual's use of the model-based reinforcement learning system, the model-free reinforcement learning system, and the frequency of switching between the two. We examined performance in subjects diagnosed with OCD, and two groups of non-affected control subjects: one with relatively high Obsessive–Compulsive Inventory-Revised (OCI-R) scores (H-OCI-R) and one with relatively low OCI-R scores (L-OCI-R) (23). We predicted that OCD and high OCI-R controls would be associated with impaired arbitration between MB and MF learning.

## 2. Materials and methods

### 2.1. Subjects

A total of 150 subjects, including 30 OCD patients and 120 healthy controls, took part in the study. The recruitment of OCD patients was *via* clinicians at the Affiliated Brain Hospital of Guangzhou Medical University. Psychiatrists used structured clinical interviews (the MINI-International Neuropsychiatric Interview, MINI) (24) to screen patients to confirm the diagnosis of obsessive–compulsive disorder [DSM-V criteria; Association AP (2013)]. In our sample, nine of the patients with OCD had a comorbid diagnosis: three with depression, six with anxiety, and three with anxiety and depression. All OCD patients had a total score of 16 or higher on the Yale–Brown Obsessive–Compulsive Scale (Y-BOCS) (25), and all patients were receiving pharmaceutical treatment (for more details, see Supplementary Table 1). Healthy controls were recruited *via* advertising at local universities. General exclusion criteria for both groups were head injury, serious medical or neurological illnesses, or substance dependence. Healthy controls were free from psychotropic medication or medical, neurological, or psychiatric conditions. Subjects received a minimum payment of 30 RMB as well as a bonus based on task performance after the experiment.

### 2.2. Clinical assessments

OCD patients completed the Y-BOCS to assess the severity of OCD symptoms (25). All subjects completed the OCI-R to assess the categories of OCD symptoms (23). Furthermore, we used the State-Trait Anxiety Inventory (STAI) to assess anxiety symptoms (27), the Beck Depression Inventory (BDI) to assess depressive symptoms (28), and the Barratt Impulsiveness Scale-11 (BIS-11) to assess impulsiveness behaviors (29).

### 2.3. Task and stimuli

**Figure 1:**
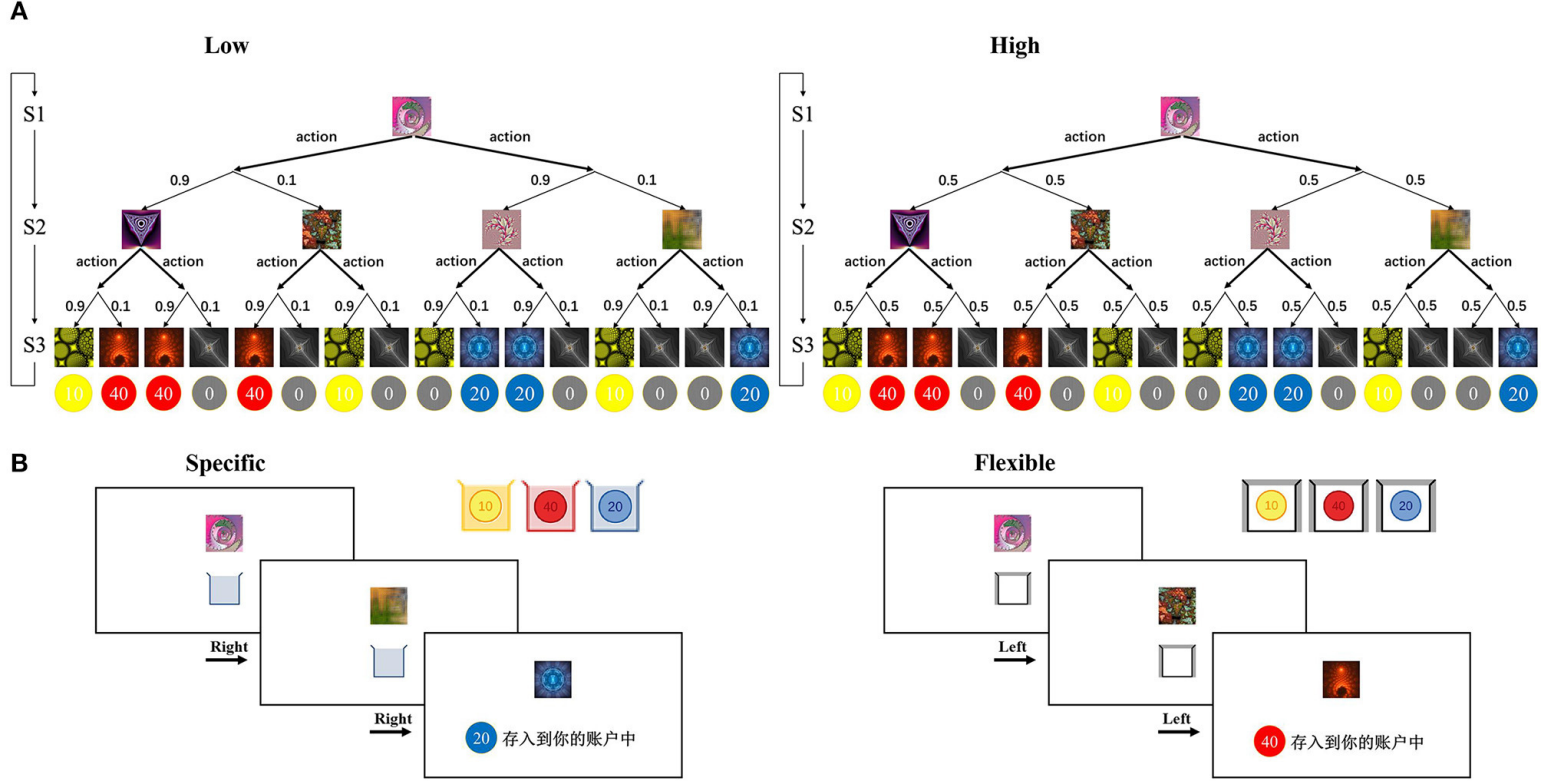
Task structure **(A)** schematic of an individual decision tree with three stages. Thick arrows indicate possible decision actions (left or right), and thin arrows indicate probabilistic transitions. The transition probability (0.9, 0.1) and (0.5, 0.5) corresponds to a low uncertainty and a high uncertainty environment, respectively. **(B)** Sample trial procedures. On each trial, the subject views a series of fractal images along with collection boxes on the computer screen. The collection boxes appear in different colors that indicate the colors of coins that will be rewarded in the specific goal condition (left) or a white collection box which will result in a reward for all colors in the flexible goal condition (right). After the second decision, the reward is displayed as the color and number of coins. Figure adapted from Weissengruber et al. (26).

We used the 2-choice, 3-stage Markov decision-making task developed by Lee et al. (9, 26). The task is illustrated in Figure 1. Subjects are required to make a series of decisions to reach end states associated with different reward values (illustrated as coins). Each subject was randomly assigned an individual decision tree (as exemplified in Figure 1A). Each trial started with the same state. After making a decision (left or right), through a probabilistic state transition (low uncertainty or high uncertainty), subjects arrived at a specific state in the next stage and then made a second decision (left or right). The trial ended with the subject winning a number of coins ranging from 0 to 40. The decision tree for each subject remained the same across the trials of the experiment so that they could learn about the transitions. Across trials, subjects had to explore and learn about the possible transformations of the tree. Subjects had 2 s to make a decision at each choice, and once they made a decision, the next state was presented 150 ms later. When the subject reached the third and final stage on each trial, the collection box disappeared and was replaced by the reward cue for the reward obtained in that state for 2 s. The reward cues were colored in yellow, red, or blue, and indicated a total number of coins of 10, 20, or 40. In addition, there were gray coins with a 0 for trials on which no reward was received.

There were two types of task conditions: the “specific” task condition and the “flexible” task condition (Figure 1B). In the specific condition, subjects were instructed that they should try to collect coins of a specific color (yellow, red, or blue) because only that color would be rewarded on the current trial. The specific condition promotes model-based control: Subjects must build an internal model of the task structure to successfully obtain rewards. In the flexible condition, subjects were instructed that all colors of coins would be rewarded. The flexible condition promotes model-free control because responding based on past reinforcement is sufficient to obtain good performance. At the beginning of each trial, the subject was cued as to whether the trial was a specific or flexible trial through the color of the collection box at the lower part of the screen (all three colors for flexible; yellow, red, or blue alone for specific trials). Subjects were instructed to collect as many coins as possible across the experiment, and the average number of coins they won was used to determine the final monetary reward, in which one coin corresponded to an additional 1 RMB.

Both conditions included two types of state transition probabilities. Blocks of low-state transition uncertainty trials extended for three to five trials in a row, whereas blocks of high-state transition uncertainty trials extended for five to seven trials in a row. This resulted in four different types of blocks (flexible or specific conditions paired with low or high uncertainty). A total of 14 of each type of block appeared in the experiment and were randomly ordered. This resulted in a total of 56 blocks which averaged a total of 280 trials. Before the actual task began, subjects completed a training phase consisting of 80 flexible trials (white collection box) followed by 20 specific trials (collection box in one of the three colors, randomly determined on each trial) to familiarize subjects with both conditions. The order of events in these training trials was the same as those in the main phase, but subjects were told that they would not receive a monetary reward for these trials. Monetary compensation was given based solely on the subjects' task performance in the main phase.

### 2.4. Computational modeling

We implemented the modeling approach presented in Weissengruber et al. (26). Model-based and model-free RL models were independently fit to each subject's data. This allowed us to quantify the preference for MB vs. MF learning by estimating the likelihood of each learning system separately for each trial. In addition, we calculated an overall arbitration score for each subject based on the ratio of MB:MF strategy use. Finally, the models allowed us to quantify how often subjects switched between the two learning systems by calculating the frequency of changes between the system with a higher likelihood on one trial and the system with a higher likelihood in the following trial.

We implemented an MF SARSA learner (30) and an MB learner (9, 26, 31). The MF learner and the MB learner calculated the state-action value using two different prediction errors, namely reward prediction errors (RPEs) and state prediction errors (SPEs), respectively. In MF learning, the experienced reward drove the learning process, whereas, in MB learning, the environment model representing the state-action-state transition probability was modified by the learning process.

The MF learner updates action values based on RPE (30). δ_*RPE*_ is the number of updates to the state-action value *Q*_*MF*_(*s, a*) for the action a in the state s. It is defined as follows:


$$\begin{array}{l} \delta_{RPE}=r\left(s^{\prime }\right)+\gamma Q_{MF}\left(s^{\prime },a^{\prime }\right)-Q_{MF}\left(s,\;a\;\right),\\ \Delta Q_{MF}\left(s,a\right)=\alpha \delta_{RPE}. \end{array}$$

Within this model, α is the learning rate (the free parameter of the model). The variables s and a are the current state and action, respectively, and s′ and a′ are the subsequent state and action. r(s′) is the reward obtained in the state s′, and γ denotes a time discount factor (31) fixed at 1.

The MB learner is based on the model developed by Lee et al. (9). It combines FORWARD learning and BACKWARD planning functions to perform state-action value updates. In the FORWARD learning function, we first define a state transition probability matrix, *T*(*s, a, s*′), which represents the probability that the agent arrives in state s′ if the agent chooses choice a in state s. The state transition probability matrix is updated based on the state prediction error (SPE) after the state transition occurs. The update functions were defined as follows:


$$\begin{array}{l} \delta_{\text{SPE}}=1-\text{T}\left({\text{s},\text{ a},\text{s}}^{\prime }\;\right),\\ \Delta \text{T}\left({\text{s},\text{a},\text{s}}^{\prime }\right)=\;\eta \delta_{\text{SPE}},\\ Q_{MB}\left(s,a\right)=\Sigma_{s^{\prime }}T\left(s,a,s^{\prime }\right)\left\{r\left(s^{\prime }\right)+max_{a^{\prime }}Q_{MB}\left(s^{\prime },\;a^{\prime }\right)\;\right\} \end{array}$$

In this model, the free parameter η represents the learning rate. The first term of the SPE is set to 1. This choice reflects the assumption that the state space is deterministic.

In the BACKWARD planning process, the FORWARD update process is repeated backward for all possible states and actions to update the value of each state (9):


$$\begin{array}{l} \text{r}\left(\text{s}\right)=\{\begin{array}{c} R\;for\;a\;goal\;state,\\ 0\;otherwise. \end{array} \end{array}$$

$$\begin{array}{lll} \text{for i}=3,2,\\ {\text{ for s}\in \text{S}}_{\text{i}-\;1}\\ \;Q_{MB}\left(s,a\right) & = & \underset{s^{\prime }}{\sum }T\left(s,a,s^{\prime }\right)\left\{r\left(S_{i}\right)+max_{a^{\prime }}Q_{MB}\left(s^{\prime },\;a^{\prime }\right)\right\},\;for\;all\;a.\\ \text{ end}\\ \text{end} \end{array}$$

where r denotes the reward value in the goal state, *S*_*i*_ is the state set of the i-th stage.

Finally, each model selects an action stochastically. We used the following softmax function (31, 32):


$$\begin{array}{l} P\left(s,a\right)=\frac{\exp\left(\tau Q\left(s,\;a\right)\right)}{\underset{b}{\sum }\exp\left(rQ\left(s,\;b\right)\;\right)}, \end{array}$$

where τ is the inverse temperature parameter, which controls the extent to which the agent chooses with a higher value action.

Following the procedure established by Lee et al. (9, 26), the Nelder–Mead simplex algorithm (33) was used to estimate the free parameters for MF and MB learners (the inverse temperature of the softmax function and the learning rate) by minimizing the negative log-likelihood −∑log(*P*(*s, a*)) of the obtained choices given the observed choices and rewards. To minimize the risk of finding a local but not a global optimum (that minimizes negative log-likelihood), we used randomly generated seed parameters and performed the optimization 200 times.

## 3. Results

### 3.1. Demographic and clinical analysis

As shown in Table 1, the OCI-R, BDI, STAI, and BIS scores of OCD patients were significantly higher than that of healthy controls. In this study, 29 healthy controls (high OCI-R score control group, H-OCI-R) with relatively high OCI-R scores (top 25%) and another 31 healthy controls (low OCI-R score control group, L-OCI-R) with relatively low OCI-R scores (bottom 25%) were selected for the analysis along with all 30 patients in the OCD group (additional analyses including the entire sample of control subjects in a single control group are shown in Supplementary Figures 1–3). The 25% prior cutoff was consistent with that used in past research (34). There were significant differences in age between the OCD group and the H-OCI-R and L-OCI-R groups, with the OCD group having a higher mean age. Because of these differences, we controlled for age as a covariate in all the analyses.

**Table 1:** Demographic and clinical variables.

	**OCD**	**HC**	**Statistics**		**H-OCI-R**	**L-OCI-R**	**Statistics (including OCD)**	
	**(*****n** =* **30)**	**(*****n** =* **120)**	χ^2^/**t**	***p***	**(*****n** =* **29)**	**(*****n** =* **31)**	χ^2^**/F**	***p***
Gender (M/F)	16/14	43/77	3.080	0.079	11/18	11/20	2.314	0.314
Age (years)	24.77 (6.87)	20.89 (2.12)	3.053^***^	< 0.001	20.14 (1.73)	21.39 (2.03)	9.403^***^	< 0.001
Y-BOCS total	23.13 (4.96)	NA	NA	NA	NA	NA	NA	NA
Obsession	12.30 (3.28)	NA	NA	NA	NA	NA	NA	NA
Compulsion	10.83 (3.56)	NA	NA	NA	NA	NA	NA	NA
OCI-R	27.33 (12.31)	18.35 (9.52)	4.347^***^	< 0.001	31.41 (5.34)	7.38 (2.73)	80.736^***^	< 0.001
BDI	20.37 (10.65)	8.31 (6.17)	5.959^***^	< 0.001	10.97 (5.80)	5.06 (4.36)	32.838^***^	< 0.001
STAI-S	55.47 (11.03)	38.08 (9.28)	8.827^***^	< 0.001	39.72 (9.29)	34.71 (7.56)	40.337^***^	< 0.001
STAI-T	59.00 (8.95)	43.42 (8.61)	8.798^***^	< 0.001	46.31 (7.82)	40.55 (7.65)	40.604^***^	< 0.001
BIS	49.81 (12.21)	37.18 (10.91)	5.532^***^	< 0.001	38.39 (10.21)	34.22 (9.94)	16.799^***^	< 0.001

OCD, obsessive–compulsive disorder; HC, healthy control; H-OCI-R, high OCI-R score control; L-OCI-R, low OCI-R score control; Including OCD, comparison of three groups including OCD; Y-BOCS, Yale–Brown Obsessive–Compulsive Scale; NA, not applicable; OCI-R, Obsessive–Compulsive Inventory-Revised; BDI, Beck Depression Inventory; STAI-S, State component of State-Trait Anxiety Inventory; STAI-T, Trait component of State-Trait Anxiety Inventory; BIS, Barratt Impulsiveness Scale.

*** < 0.001.

### 3.2. Task performance analysis

**Figure 2:**
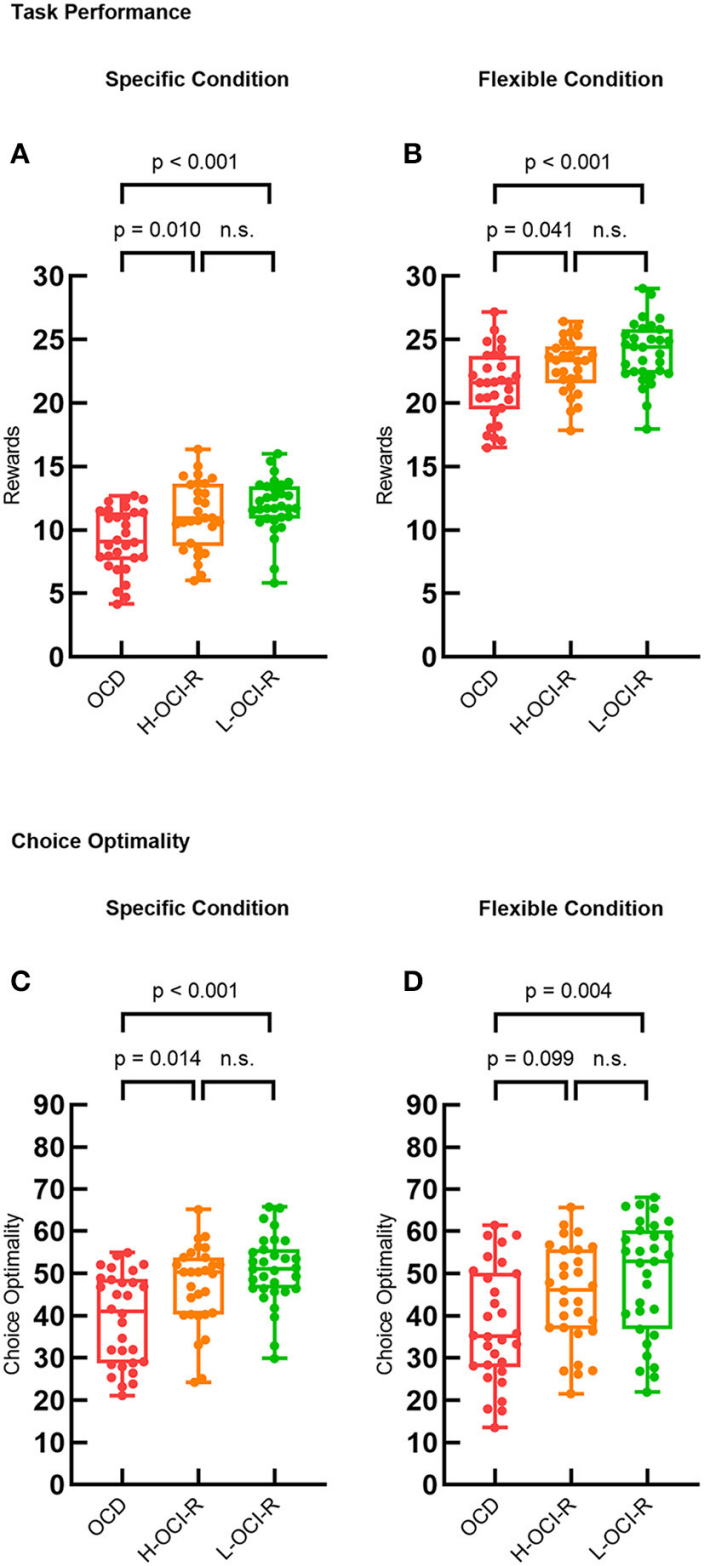
Task performance. Task performance measured as the average reward (mean number of coins) earned on each trial across the three groups in **(A)** the specific condition and **(B)** the flexible condition. Choice optimality across the three groups in **(C)** the specific condition and **(D)** the flexible condition. OCD, obsessive–compulsive disorder; H-OCI-R, high OCI-R score control; L-OCI-R, low OCI-R score control.

We used one-way ANOVA to examine the performance of the three subject groups in the specific and flexible conditions separately. The first dependent variable was the mean number of coins earned per trial, which was used as a measure of overall task performance. There were significant differences in task performance between groups in the specific condition (F_2, 86_ = 7.87, *p* = 0.001). *Post hoc* analysis demonstrated that the OCD group (*M* = 9.23, *SD* = 2.48) performed worse than the L-OCI-R group (*M* = 11.91, *SD* = 2.13, *p* < 0.001) and the H-OCI-R group (*M* = 11.17, *SD* = 2.69, *p* = 0.010). We found no significant difference between the H-OCI-R and L-OCI-R groups (*p* = 0.236; Figure 2A). In the flexible condition, we also found group differences in the number of points the subjects earned (F_2, 86_ = 7.32, *p* = 0.001). *Post hoc* analysis of the differences between groups further revealed that the OCD group (*M* = 21.43, *SD* = 2.74) was inferior to the L-OCI-R group (*M* = 24.00, *SD* = 2.42, *p* < 0.001) and the H-OCI-R group (*M* = 22.94, *SD* = 2.11, *p* = 0.041) in task performance. There was no significant difference between the H-OCI-R and L-OCI-R groups (*p* = 0.095; Figure 2B).

We also examined the dependent variable of choice optimality. Choice optimality was defined as the percentage of trials on which subjects made the optimal sequence of decisions for the trial (that is, the decisions that resulted in the highest possible reward). There were group differences in the specific condition (F_2, 86_ = 8.67, *p* < 0.001) and the flexible condition (F_2, 86_ = 4.41, p = 0.015). *Post-hoc* tests showed that compared with the L-OCI-R group (*M* = 50.79, *SD* = 8.29, *p* < 0.001) and the H-OCI-R group (*M* = 47.08, *SD* = 9.71, *p* = 0.014), the OCD group (*M* = 39.26, *SD* = 10.85) had a lower choice optimality in the specific condition. We found no significant difference between the H-OCI-R and L-OCI-R groups (*p* = 0.120; Figure 2C). Furthermore, compared with the L-OCI-R group (*M* = 48.93, *SD* = 13.79), the OCD group (*M* = 37.70, *SD* = 13.69) also had a lower choice optimality in the flexible condition (*p* = 0.004; Figure 2D).

### 3.3. Learning strategy analysis

**Figure 3:**
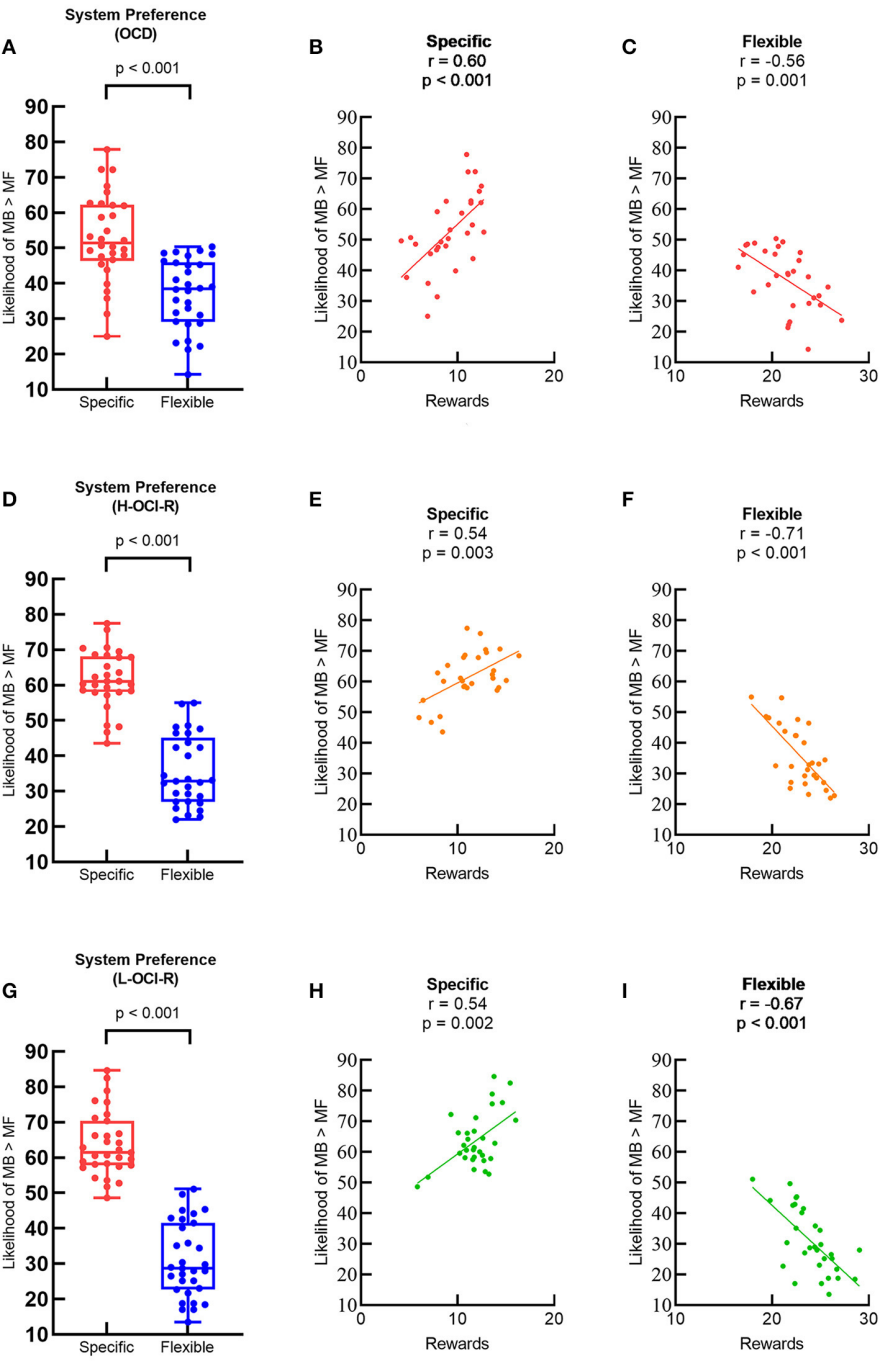
Preference for MB control (% of choices where the MB learning has a higher likelihood than the MF learning) between different task conditions in **(A)** the OCD group, **(D)** the H-OCI-R group, and **(G)** the L-OCI-R group. Task performance and preference for MB control were positively correlated in specific blocks in **(B)** the OCD group, **(E)** the H-OCI-R group, and **(H)** the L-OCI-R group but negatively correlated in flexible blocks in **(C)** the OCD group, **(F)** the H-OCI-R group, and **(I)** the L-OCI-R group. OCD, obsessive–compulsive disorder; H-OCI-R, high OCI-R score control; L-OCI-R, low OCI-R score control.

Individual learning strategies were characterized using system preference and system switching parameters. We first examined system preference using separate paired-sample *t*-tests for each group of subjects as a manipulation check to confirm that different task conditions promoted different learning strategies. According to the logic of the experimental paradigm, in the specific goal condition, the trial-by-trial goal changes necessitate goal-directed learning (must use the MB strategy to do well), while in the flexible goal condition, subjects would prefer cost-effective learning strategies (just using the MF strategy is sufficient). Consistent with past research (26), MB systems were preferred more often on specific trials than on flexible trials for all groups of subjects, indicating that overall our task manipulation and computational modeling approach was successful. Figure 3A shows that preference in the OCD group for MB control was greater on specific trails than on flexible trails (t_29_ = 5.48, *p* < 0.001). The OCD group's task performance on specific trails was positively correlated with a stronger preference for MB learning (r_28_ = 0.60, *p* < 0.001; Figure 3B), but performance on flexible trials was negatively correlated with a stronger preference for MB learning (r_28_ = −0.56, *p* = 0.001; Figure 3C). The same patterns were also found in the H-OCI-R group (Figures 3D–F) and the L-OCI-R group (Figures 3G–I).

**Figure 4:**
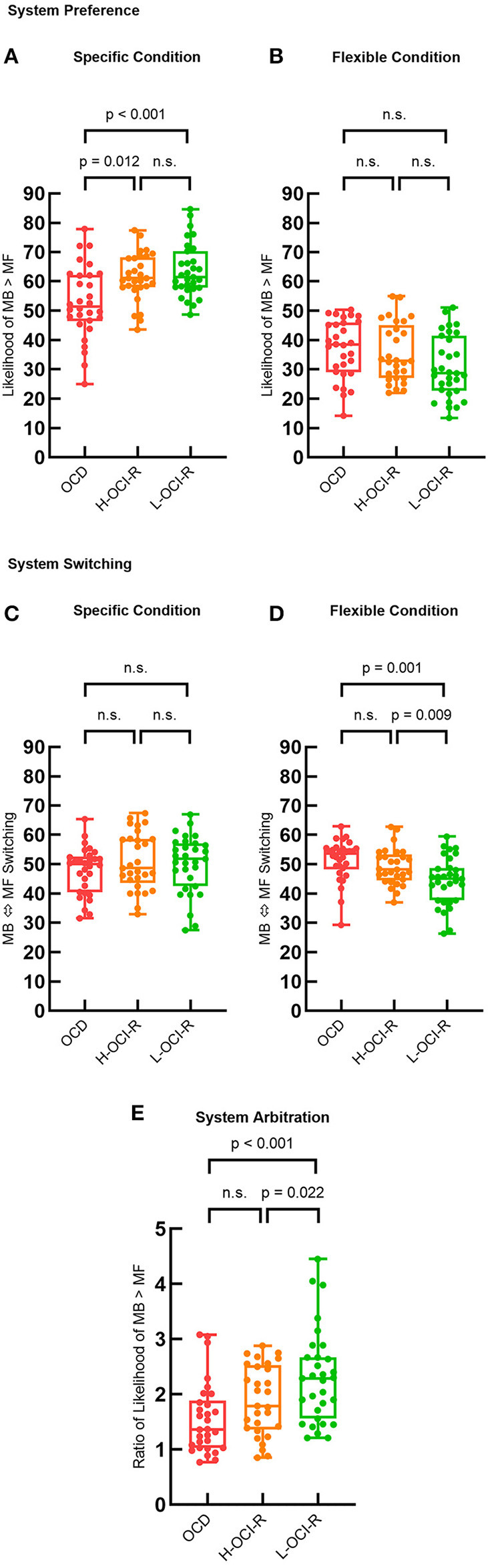
Group differences in learning strategies. System preference parameters across the three groups in **(A)** the specific condition and **(B)** the flexible condition. System switching (% of trials where the dominant system switches from that used in the previous trial) across the three groups in **(C)** the specific condition and **(D)** the flexible condition. **(E)** System arbitration parameter (ratio of system preference across conditions) across subject groups. OCD, obsessive–compulsive disorder; H-OCI-R, high OCI-R score control; L-OCI-R, low OCI-R score control.

Then, we compared system strategy preferences and appropriate matches with task conditions across groups, as illustrated in Figure 4. The specific condition is best learned *via* MB strategies, whereas the flexible condition is best learned *via* MF strategies. One-way ANOVA showed that there were significant differences in system preference across groups of subjects in specific conditions (F_2, 86_ = 6.93, *p* = 0.002), which is best supported by MB learning. *Post-hoc* analysis suggested that the OCD group (*M* = 52.84, *SD* = 12.32) showed a greater inappropriate preference for MF learning than the L-OCI-R group (*M* = 63.72, *SD* = 9.04, *p* < 0.001) and the H-OCI-R group (*M* = 61.51, *SD* = 8.23, *p* = 0.012) in the specific condition. We did not find a significant difference between the H-OCI-R group and the L-OCI-R group (*p* = 0.327; Figure 4A). In the flexible condition, best supported by MF learning, we did not find differences in system preference across groups (F_2, 86_ = 2.61, *p* = 0.079; Figure 4B). Overall, the OCD group showed worse appropriate matching of learning strategy with task conditions.

We then examined system switching rates. There were no significant differences in the rate of system switching between groups in the specific condition (F_2, 86_ = 0.21, *p* = 0.808; Figure 4C). However, we found significant differences between groups in the flexible condition (F_2, 86_ = 7.23, *p* = 0.001). Compared with the L-OCI-R group (*M* = 44.25, *SD* = 8.22), the OCD group (*M* = 51.46, *SD* = 6.96) showed an increased rate of switching across trials (*p* = 0.001). Furthermore, compared with the L-OCI-R group, the H-OCI-R group (*M* = 49.16, *SD* = 6.15) had a higher system switching rate (*p* = 0.009; Figure 4D). This indicates that these two groups found it difficult to maintain a consistent strategy, whether it was MB or MF control, under flexible conditions.

To compare the overall likelihood of appropriate system choice across groups, we calculated a measure of system arbitration. This was defined as the ratio of the subjects' system preference for MB in the two different conditions (specific condition/flexible condition). We then compared the system arbitration parameter across groups using a one-way ANOVA (F_2, 86_ = 7.02, *p* = 0.001). The results showed reduced system arbitration in the OCD group (*M* = 1.56, *SD* = 0.64) than in the L-OCI-R group (*M* = 2.31, *SD* = 0.85, *p* < 0.001), with OCD and H-OCI-R groups not differing significantly from each other (*p* = 0.188). In addition, compared with the L-OCI-R group, the H-OCI-R group (*M* = 1.89, *SD* = 0.63) had a lower system arbitration (*p* = 0.022; Figure 4E).

### 3.4. Specificity to OC symptoms

Past research has found that depressive symptoms affect the arbitration process (35). To control for whether the relationship between arbitration and OCD symptoms was due to anxiety, depression, or impulsiveness, we also performed analyses in which we included the three as covariates. The covariance analyses controlling for state anxiety, trait anxiety, depression, and impulsiveness showed that in the specific condition, the OCD patients still showed less model-based strategy choice than the H-OCI-R group (*p* = 0.033) and the L-OCI-R group (*p* = 0.012); in the flexible condition, the OCD patients (*p* = 0.001) and the H-OCI-R group (*p* = 0.004) still switched more often between systems than the L-OCI-R group, and the OCD patients (*p* = 0.005) and the H-OCI-R group (*p* = 0.017) still had a lower system arbitration parameter than the L-OCI-R group. In light of these findings, we conclude that arbitration process impairment is specific to compulsion rather than being due to depression, anxiety, or impulsiveness.

## 4. Discussion

Our findings provided evidence for the impairment of the arbitration system in both OCD patients and non-clinical subjects with high OCI-R scores (H-OCI-R). We found two aspects of arbitration were impaired. First, we measured preference for MB and MF systems during tasks differing in whether MB or MF strategies were optimal and found suboptimal preferences. In the specific condition, best learned *via* the MB system, the OCD group showed a lower preference for MB than the H-OCI-R and the L-OCI-R groups. Second, we examined system switching and found that both OCD and H-OCI-R groups switched more frequently in the flexible condition best learned by the MF system, which indicates less stable strategy use potentially resulting from impaired arbitration. In summary, OCD patients showed an impaired ability to change preferences for behavioral control systems based on environmental demands.

OCD patients showed different impairments in the specific task and the flexible task, consistent with previous studies finding that the arbitrator functions differently in different conditions (26). According to Kim and collaborators, healthy individuals increase MB control when the environment becomes complex (36). We showed that under the more complex specific task condition, OCD patients were less biased toward MB learning and lacked proper arbitration. Consistent with this, past research found that OCD groups use fewer model-based strategies in a 2-step RL task similar to the specific condition in our study (12–15).

Overall, we found a greater ability of the L-OCI-R group than the H-OCI-R and OCD groups to choose to use the strategy best suited for each task. This agrees with previous studies finding greater flexibility for L-OCI-R and inflexibility in OCD (37). In our task, as shown in Figure 3, specific task conditions favor MB learning, whereas flexible task conditions favor MF learning. Therefore, the optimal strategy may be to maximize the use of MB learning in specific trials, while relying on the MF strategy with less cognitive resource consumption in flexible trials. In the flexible condition when the environment becomes simple, both the OCD and the H-OCI-R groups' ability to flexibly adapt to environmental demands was impaired (mainly manifested in the inability to maintain the use of MF learning). Similarly, past research found that OCD patients have diminished stimulus stickiness (increased switching) when learning optimal behavior in a stable environment (38). All these indicate that individuals with compulsive behaviors are impaired in their ability to choose the most appropriate system.

The impairment in arbitration we observed in OCD raises the question of the neural systems underlying this impairment. Functional MRI research in healthy subjects using this task found that assessment of the reliability of the dual systems by the arbitration system mainly involves the anterior cingulate cortex (ACC) and the ventrolateral prefrontal cortex (vlPFC) (9, 11). Furthermore, a neurostimulation study targeting the vlPFC indicated that individual arbitration of dual systems is mainly achieved by inhibiting or releasing model-free system activation, similar to a “valve” (26). Both the ACC and vlPFC are components within the cortical–striatal–thalamic–cortical circuits implicated in OCD (39). The ACC participates in conflict monitoring and behavioral outcome assessment in the process of information processing (40), weighing cognitive loss and gain to allocate cognitive control over behavior, and monitoring conflict between goal-directed and habitual behavior learning (41). Previous studies found that patients with OCD have abnormal monitoring of conflicts and abnormal activation of ACC (42). The research found that increased activation of the putamen during OCD symptom provocation was correlated with increased activation of the ACC (18). In addition, resting state functional connectivity studies of patients with OCD found that the ACC-related intrinsic connectivity was abnormal (43). These studies indicate that ACC abnormalities may cause OCD patients to fail to successfully arbitrate between conflicting systems and fail to achieve the purpose of selecting the most appropriate behavioral learning system.

Arbitration has also been linked to the vlPFC. A functional MRI study found recruitment of the vlPFC for arbitration (9). A neuromodulation study found that enhancing vlPFC activity in the specific condition increased preference for the model-based system, and inhibiting vlPFC activity in flexible conditions reduced system switching, the results which are both consistent with vlPFC contributing to the arbitration process (26). A further study found that vlPFC is important for prediction error baseline adjustment, a critical aspect of arbitration (44). The vlPFC is located in the ventral cognitive subcircuit of the cortical–striatal–thalamic–cortical circuit and is related to Abnormal inhibitory control in OCD patients (39). This anomaly is considered a stable trait of OCD (45).

This study has some limitations that should be taken into consideration. First, age, depression, anxiety, and impulsiveness were not matched between the OCD and control groups. However, age, depression, state anxiety, trait anxiety, and impulsiveness were controlled for as covariates in all analyses, and none affected the overall pattern of results. Second, the study did not control for cognitive indicators that may lead to differences in task performance, such as IQ and working memory. Third, a recent study showed that participants' proneness to misunderstanding of instructions leads to inaccurate MB/MF estimates (46), indicating that there are limitations in the use of sequential decision tasks.

Our study is the first to experimentally explore the role of the arbitration process in OCD. We found that arbitration was impaired in patients with OCD and also in controls with high OCI-R scores, both in terms of impaired ability to select the most appropriate strategy and to maintain an appropriate strategy over trials. These results may contribute to a greater understanding of how impairments in instrumental learning may underlie the symptoms of OCD.

## Data availability statement

The original contributions presented in the study are included in the article/Supplementary material, further inquiries can be directed to the corresponding authors.

## Ethics statement

The studies involving human participants were reviewed and approved by the South China Normal University Research Ethics Committee. The patients/participants provided their written informed consent to participate in this study.

## Author contributions

CS, QC, and ZP: conceptualization. ZR, DK, SL, QC, and ZP: methodology. ZR: formal analysis, investigation, and writing – original draft. QY: resources. CS, DK, SL, QC, and ZP: writing – reviewing and editing. QC and ZP: supervision and funding acquisition. All authors contributed to the manuscript and approved the submitted version.
