# Supplementary material for: Impairment of arbitration between model-based and model-free reinforcement learning in obsessive–compulsive disorder
Source: Front Psychiatry. 2023 May 26;14:1162800. doi: 10.3389/fpsyt.2023.1162800 (PMC10250695; doi:10.3389/fpsyt.2023.1162800)
Supplement: Supplementary file 1 [file Table_1.DOCX]

Supplementary Material

Impairment of arbitration between model-based and model-free reinforcement learning in obsessive-compulsive disorder

Zhongqiang Ruan, Carol A. Seger, Qiong Yang, Dongjae Kim, Sang Wan Lee, Qi Chen*, Ziwen Peng*

*** Correspondence:**

Ziwen Peng

pengzw@m.scnu.edu.cn

Qi Chen

chenqi@szu.edu.cn

**Table S1.** Details of pharmaceutical treatments for OCD patients

| Treatment | Number of cases | Average dosage (mg) |
| --- | --- | --- |
| fluvoxamine | 14 | 200 |
| fluvoxamine + quetiapine | 2 | 150 + 200 |
| fluvoxamine + tandospirone | 2 | 150 + 40 |
| sertraline | 8 | 150 |
| sertraline + lithium carbonate | 1 | 150 + 500 |
| escitalopram | 2 | 40 |
| paroxetine + sodium valproate | 1 | 40 + 500 |

**
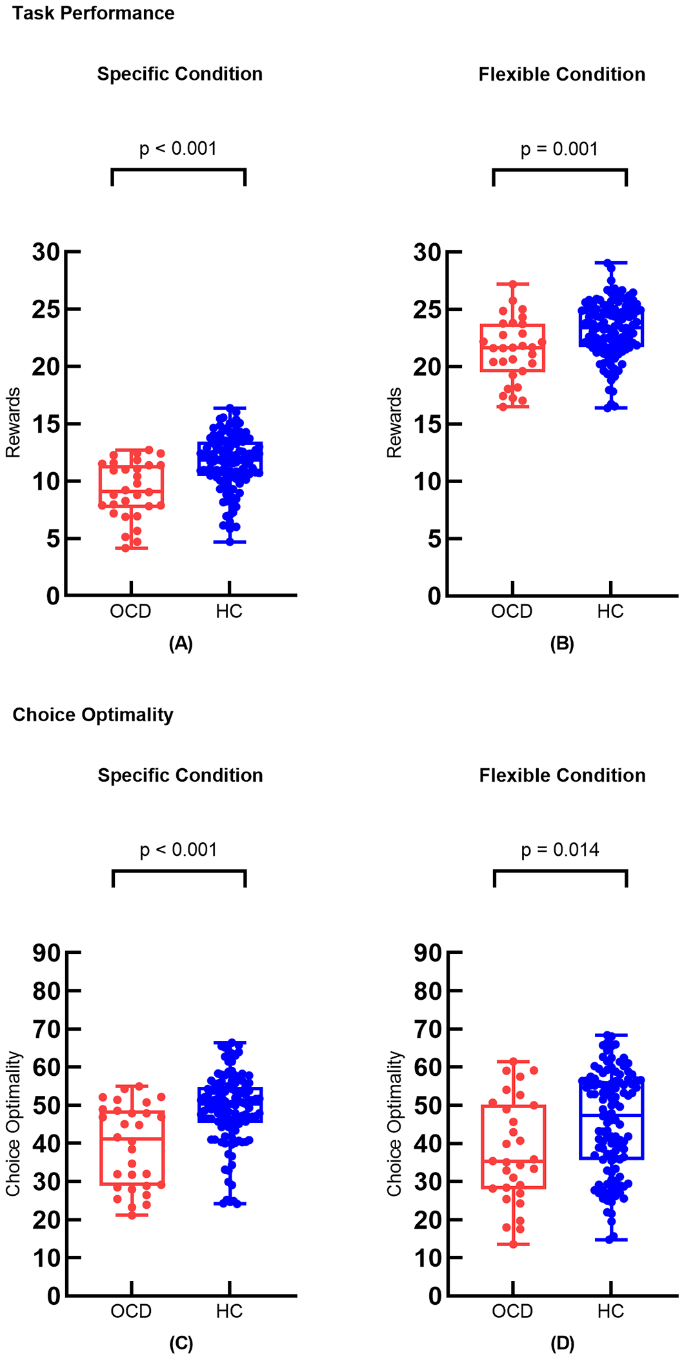
**

**Figure S1.** **Task performance comparison of OCD with all control participants.** The task performance of the OCD group was significantly lower than that of the HC group in (A) the specific condition and (B) the flexible condition. The OCD group also showed significantly lower choice optimality in (C) the specific condition and (D) the flexible condition compared to the HC group. OCD: Obsessive Compulsive Disorder, HC: healthy control.


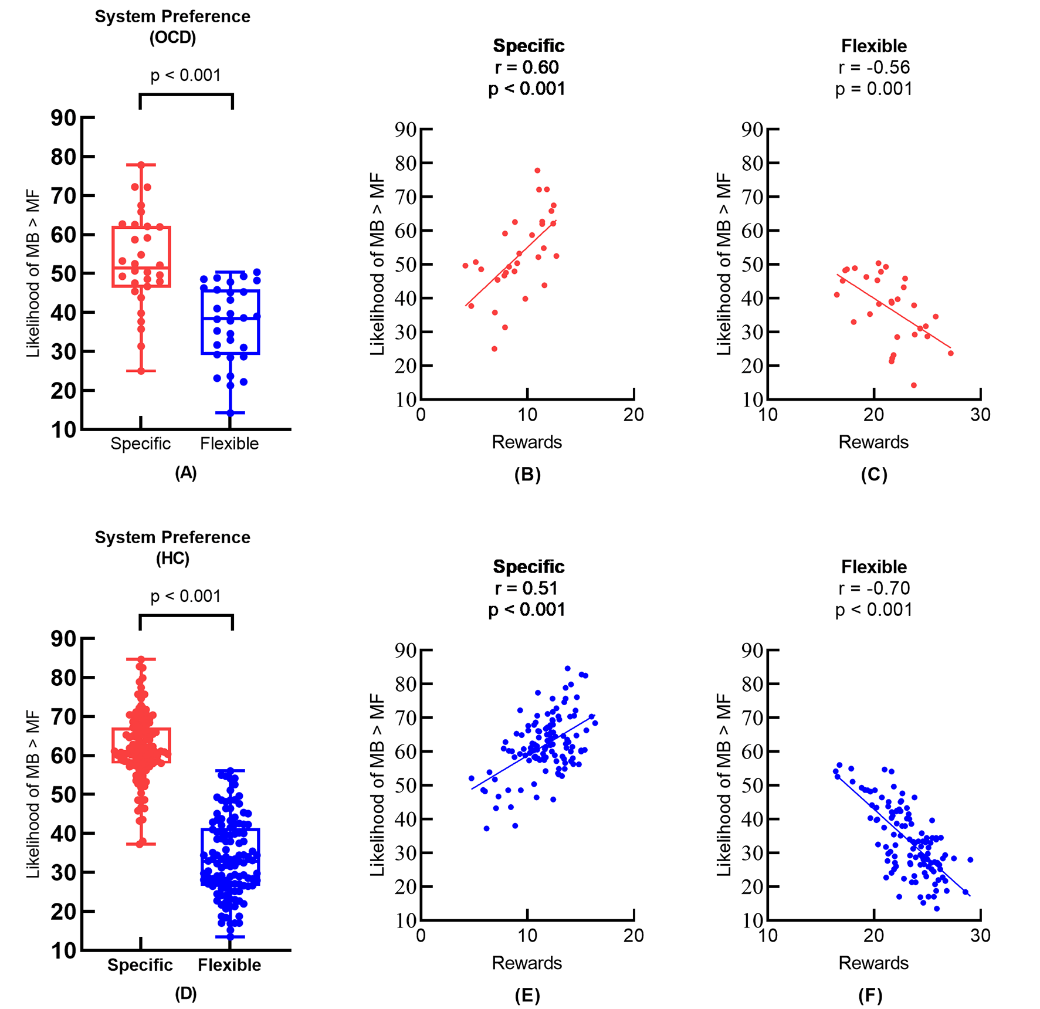


**Figure S2. System preference comparison of OCD with all control participants.** Both (A) OCD group and (D) HC group preferred MB control in specific trials. Task performance and preference for MB control was positively correlated in specific blocks in (B) the OCD group and (E) the HC group, but negatively correlated in flexible blocks in (C) the OCD group, and (F) the HC group. OCD: Obsessive Compulsive Disorder, HC: healthy control.


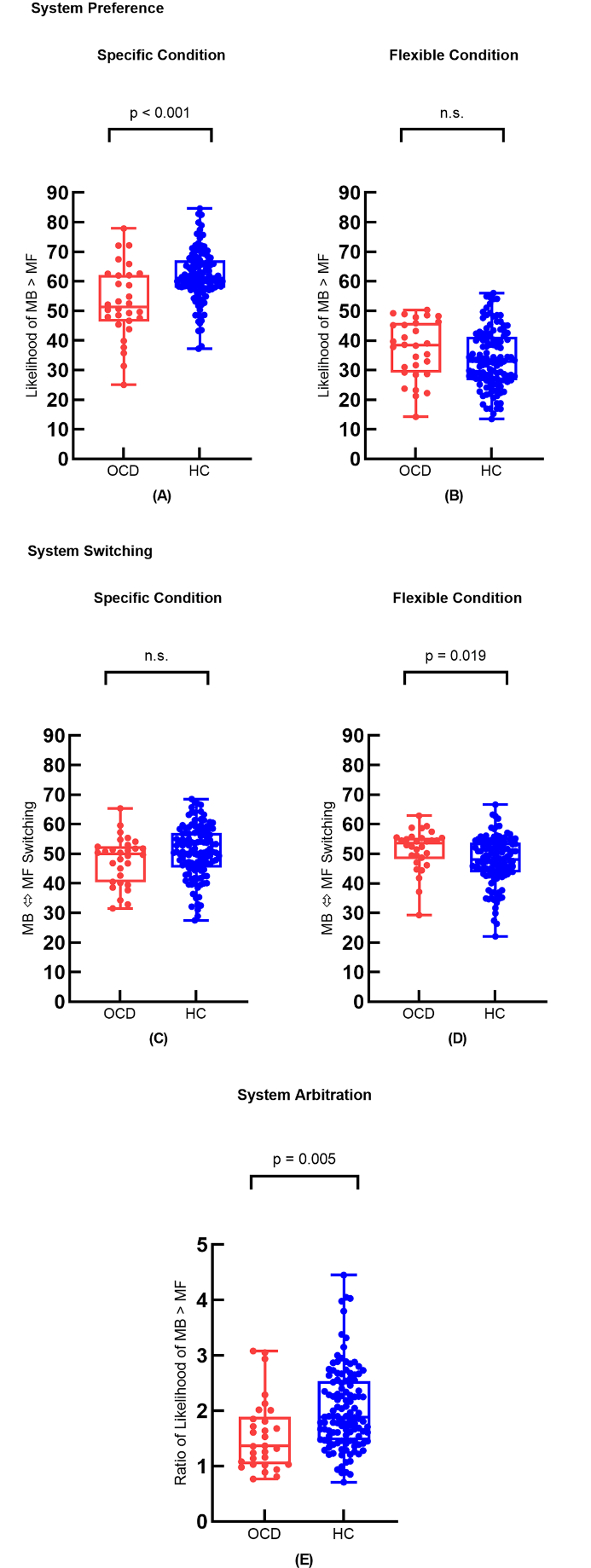


**Figure S3.** **Group differences** **in learning** **strategies between OCD and all control participants**. In (A) the specific condition, the OCD group used the MB strategy less than the HC group, but there was no significant difference between the two in (B) the flexible condition. In (C) the specific condition, there is no significant difference between the two, but in (D) the flexible conditions, the OCD group switched between strategies more frequently than the HC group. (E) The system arbitration parameters of the OCD group were significantly lower than those of the HC group. OCD: Obsessive Compulsive Disorder, HC: healthy control.
